# Supplementary material for: Human apical-out nasal organoids reveal an essential role of matrix metalloproteinases in airway epithelial differentiation
Source: Nat Commun. 2024 Jan 2;15:143. doi: 10.1038/s41467-023-44488-1 (PMC10762242; doi:10.1038/s41467-023-44488-1)
Supplement: Supplementary file 1 — Supplementary Information [file 41467_2023_44488_MOESM1_ESM.pdf]

Supplementary Information

1. Supplementary Table

Supplementary Table 1: Expression difference of MMP genes in differentiation versus proliferation in organoid cultures (based on RNA-seq data)

Gene	D17 vs D10		D24 vs D10	
	Log2FC	p-value	Log2FC	p-value
MMP9	6.149250208	1.21E-17	7.161855	2.27E-23
MMP7	2.795281287	4.86E-08	5.84972	2.11E-31
MMP10	5.566200532	1.8E-07	6.978591	5.95E-11
MMP13	7.609526659	8.23E-06	8.812906	2.39E-07
MMP2	4.22812456	1.74E-10	4.369371	4.2E-11
MMP14	-1.05929149	1.03E-09	-1.87062	4.48E-27
MMP12	3.890111719	0.003104	6.05556	1.65E-06
MMP3	4.851403243	0.000473	4.753154	0.000615
MMP1	-2.554132267	0.01109	-1.39075	0.164866
MMP28	-0.86416012	0.002851	-2.123811927	2.45E-13
MMP11	-0.080419031	0.924426	0.294757	0.726241
MMP15	0.515518697	0.002095	-0.21514	0.201599
MMP17	-0.206603233	0.670881	-0.74637	0.131757
MMP19	0.645270932	0.285933	0.227761	0.709265
MMP21	1.365845309	0.205578	1.804288	0.091732
MMP23A	4.344232363	0.094329	4.789229955	0.064062
MMP23B	-0.545859151	0.341652	0.484399479	0.381963
MMP24	-0.259112578	0.458207	0.683137388	0.043737
MMP25	-0.157182368	0.640551	-0.411920208	0.223095

2. Supplementary figures and legends

Supplementary Figure 1

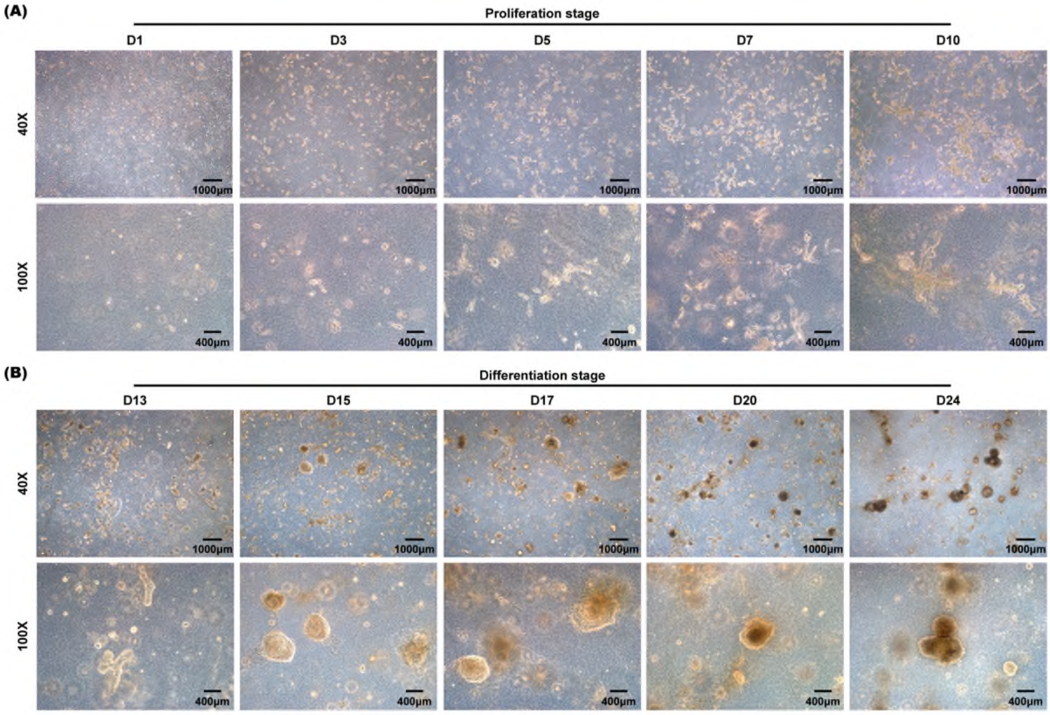

Supplementary Figure 1 Bright field microscopic views of hANOs during proliferation and differentiation time points. Magnification is 40x and 100x respectively.

Supplementary Figure 2

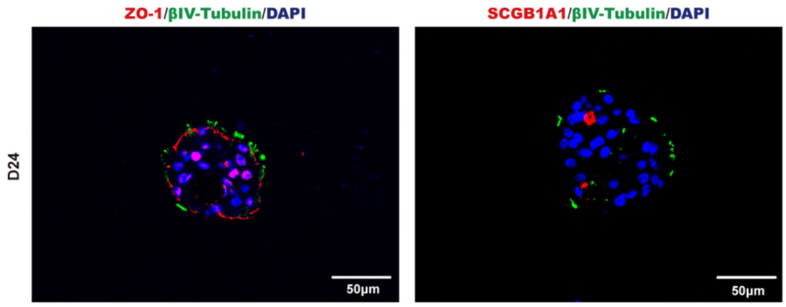

Supplementary Figure 2 Cross-sectional images of ZO-1, βIV-tubulin, and SCGB1A1 immunofluorescence staining (by confocal microscopy) in hANOs at differentiated stage (Day 24).

Supplementary Figure 3

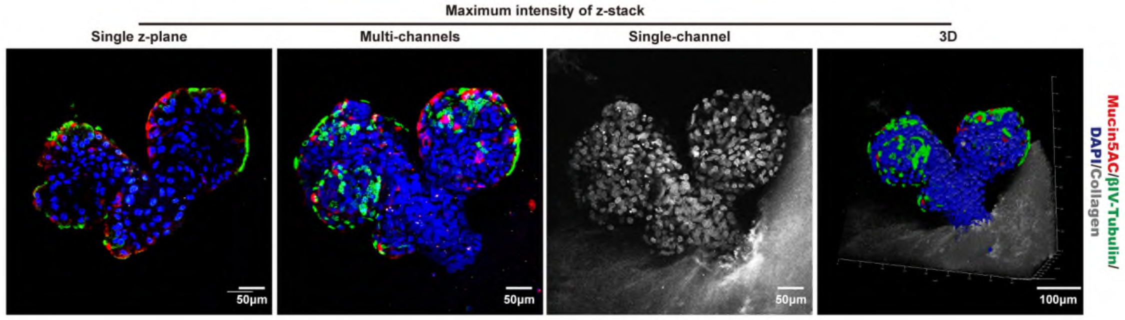

Supplementary Figure 3 3D reconstruction images of βIV-tubulin and MUC5AC immunofluorescence staining (by multiphoton microscopy) in hANOs at differentiated stage (Day 24).

Supplementary Figure 4

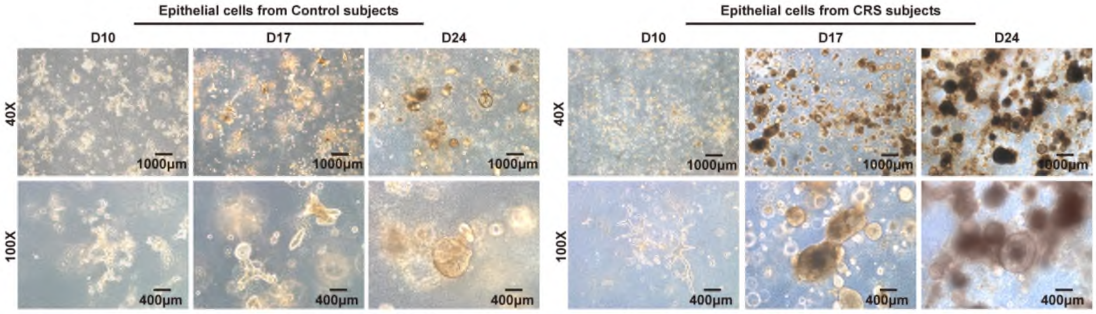

Supplementary Figure 4 Bright field microscopic views of hANOs during proliferation and differentiation time points. The epithelial cells were obtained from nasal mucosa using brushing method. Magnification is 40x and 100x respectively.

Supplementary Figure 5

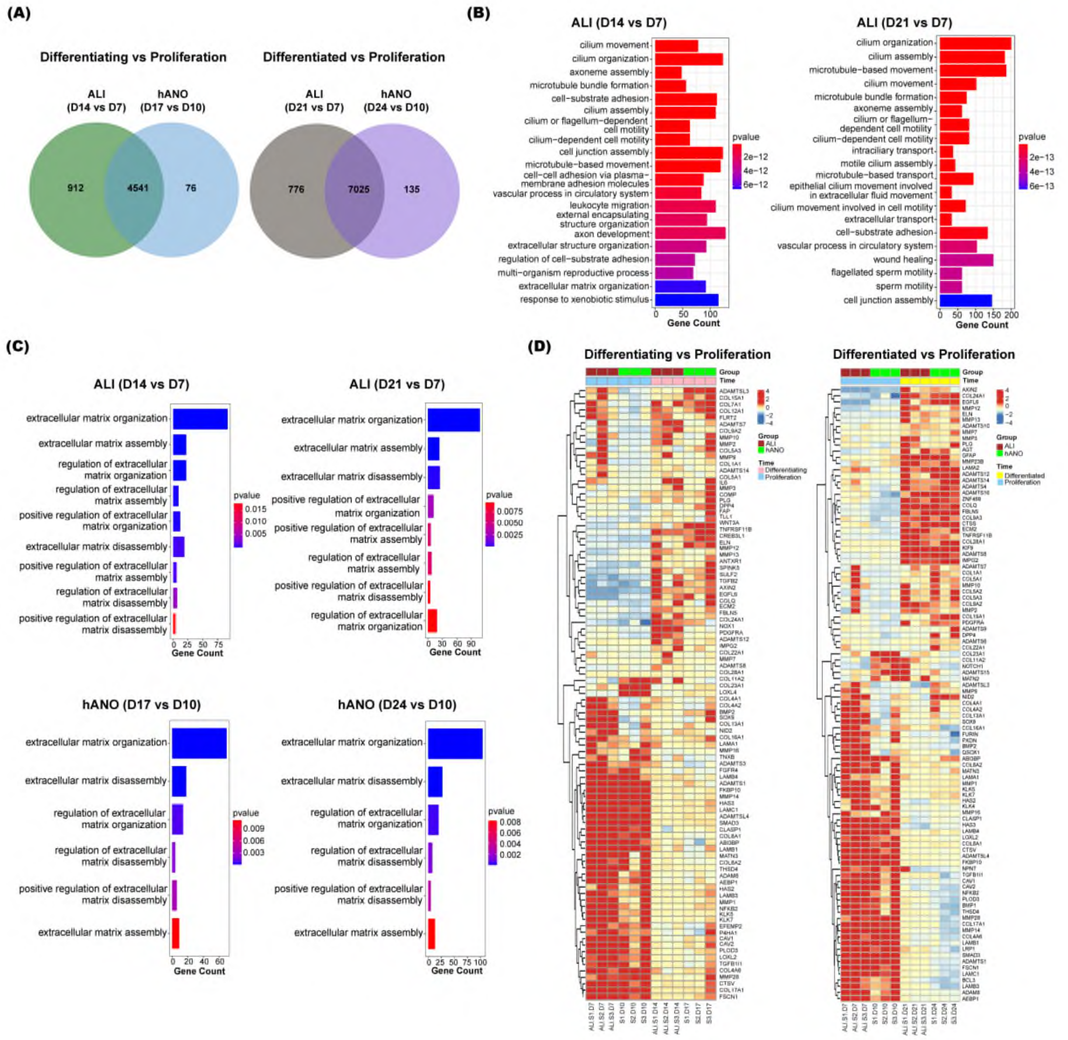

Supplementary Figure 5 Comparison of molecular characteristics between hANO and ALI model. (A) Venn diagram analysis shows the overlapping genes between the two culture systems. (B) Biological process analysis demonstrates the relevant functional terms in different differentiation stages of hANO and ALI cultures. (C) Functional terms related to extracellular matrix (ECM) organization of these two models are listed in parallel. (D) Heatmap demonstrates genes associated with ECM organization in proliferation and differentiation stages of hANO and ALI cultures. n = 3 experiment per condition; the experiment was independently performed in three organoid lines from three different donors.

Supplementary Figure 6

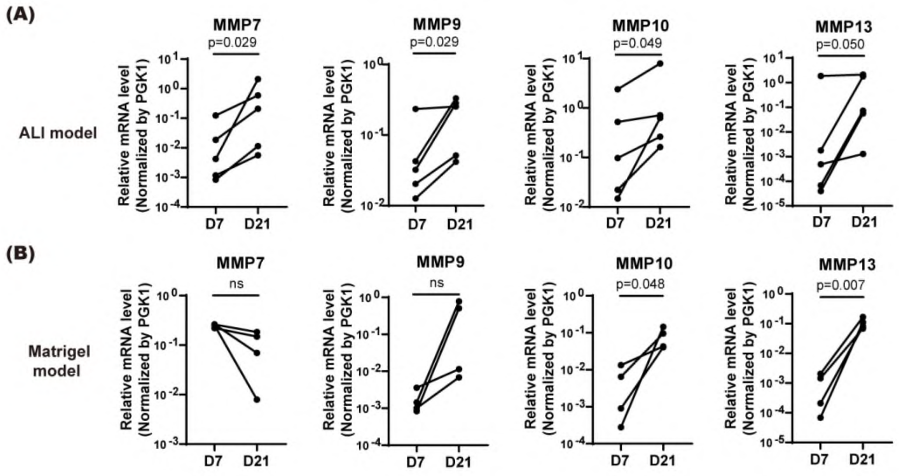

Supplementary Figure 6 mRNA levels of MMP7, MMP9, MMP10, and MMP13 were analyzed in ALI (A) and Matrigel model (B) at proliferation (D7) and differentiation (D21) stages by qPCR assays. For ALI model, n = 5 experiments per condition; the experiment was independently performed in five ALI cultures from five different donors; for Matrigel model, n = 4 experiments per condition; the experiment was independently performed in two organoid lines from two different donors. The paired t test was used in comparison analysis in (A) and (B).

Supplementary Figure 7

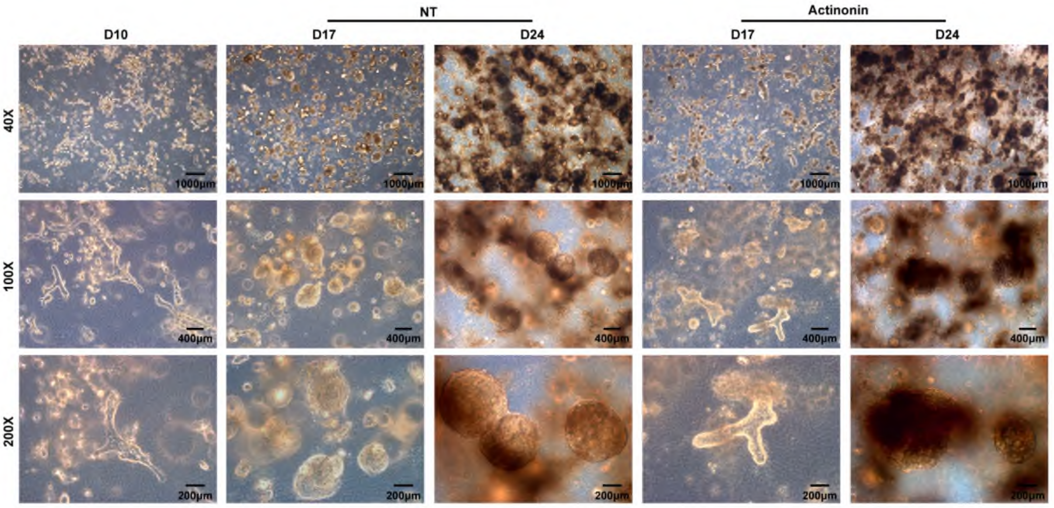

Supplementary Figure 7 Bright field microscopic views of hANOs treated with and without actinonin during differentiation stages. Magnification is 40x, 100x, and 200x

respectively.

Supplementary Figure 8

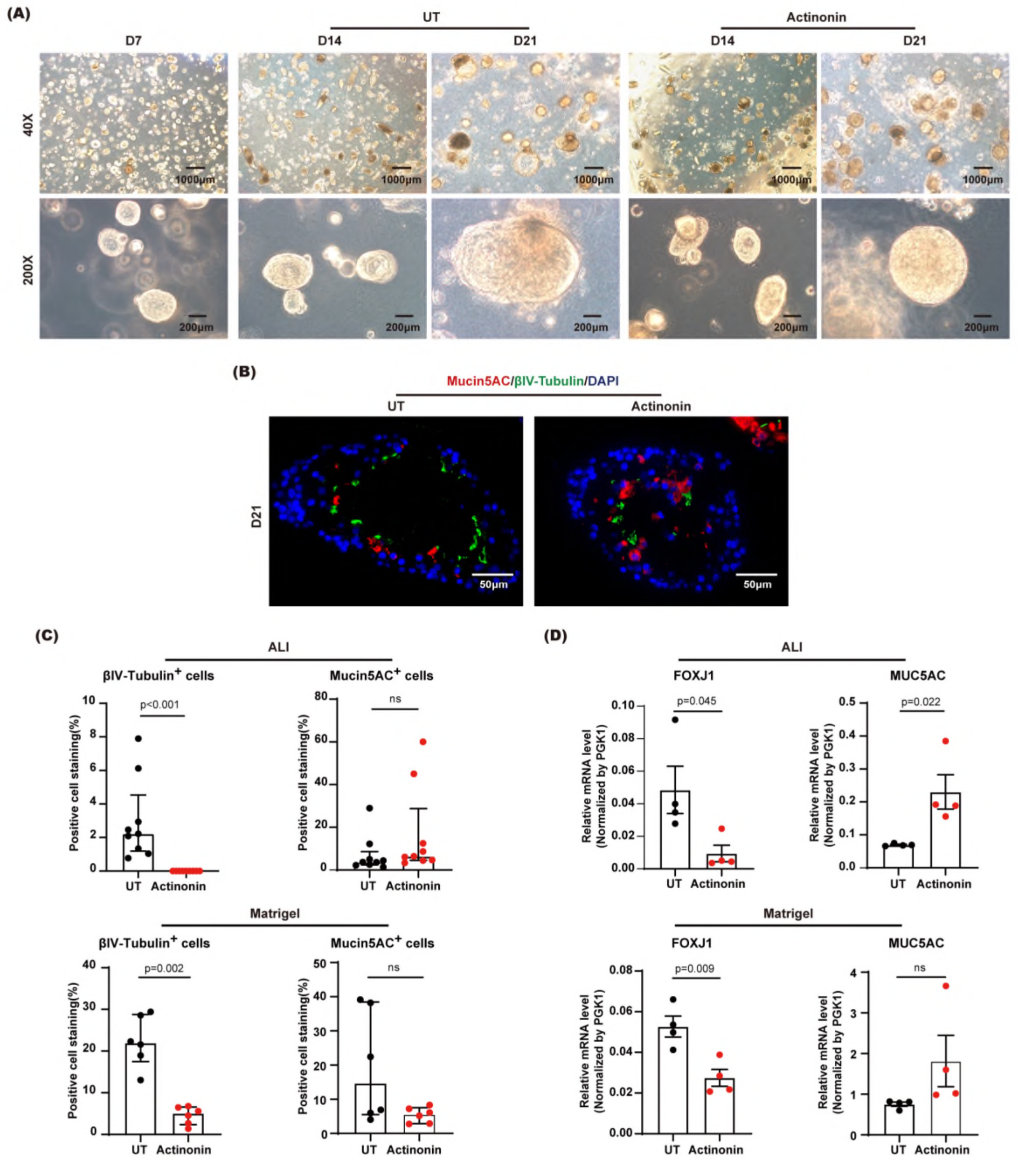

Supplementary Figure 8 Characterization of ALI cultures and Matrigel-based apical-in organoids treated with MMP inhibitor. (A) Bright field microscopic views of apical-in organoids treated with and without actinonin during differentiation stages. Magnification is 40x and 200x respectively. (B) Representative pictures of candidate cellular markers (βIV-tubulin and MUC5AC) of apical-in organoids (cross-sections of organoids captured by confocal microscopy) in differentiated organoids (D21) treated with actinonin versus untreated organoids. (C-D) βIV-tubulin+ cells, MUC5AC+ cells

and mRNA expression of Foxj1 and MUC5AC were compared in actinonin-treated to untreated ALI or apical-in organoids; for positive cell analysis, data present the median with interquartile, n = 6 and 9 experiments per condition in organoids and ALI model respectively; for qPCR analysis, data present the mean with SEM, n= 4 experiments per condition in organoids and ALI model respectively; the ALI experiment was independently performed in three cultures from three different donors, while the apical-in organoid experiment was independently performed in two organoid lines from two different donors. The Mann-Whitney test was used in comparison analysis in *(C)*; the unpaired t test was used in comparison analysis in *(D)*; ns, not significant.

Supplementary Figure 9

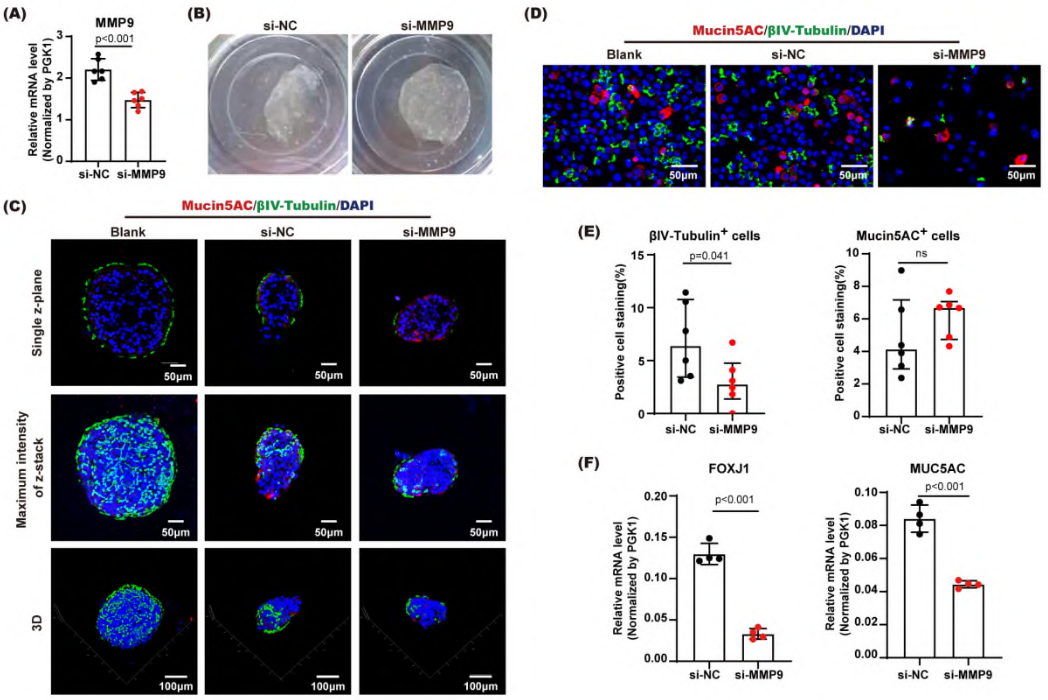

Supplementary Figure 9 Characterization of hANOs treated with MMP9 siRNA. (A) mRNA expression levels of MMP9 in hANOs treated with MMP9 siRNA versus negative control siRNA. (B) Gel degradation in differentiated (D24) hANOs treated with MMP9 siRNA versus negative control siRNA. (C-D) Representative pictures of candidate cellular markers (βIV-tubulin and MUC5AC) of hANO (by multiphoton microscopy and immunofluorescent microscopy) in differentiated hANOs (D24)

treated with siRNA-MMP9 compared to negative control siRNA. (E-F) βIV-tubulin+ cells, MUC5AC+ cells and mRNA expression of Foxj1 and MUC5AC were compared in siRNA-MMP9 to negative control siRNA; for positive cell analysis, data present the median with interquartile, n = 6 experiments per condition; for qPCR analysis, data present the median with interquartile, n=6 experiments per condition; the above two experiments were independently performed in three organoid lines from three different donors. The unpaired t test was used in comparison analysis in *(A)* and *(F)*; the Mann-Whitney test was performed in *(E)*. ns, not significant.
