# Supplementary material for: Human apical-out nasal organoids reveal an essential role of matrix metalloproteinases in airway epithelial differentiation
Source: Nat Commun. 2024 Jan 2;15:143. doi: 10.1038/s41467-023-44488-1 (PMC10762242; doi:10.1038/s41467-023-44488-1)
Supplement: Supplementary file 3 — Description of Additional Supplementary Files [file 41467_2023_44488_MOESM3_ESM.pdf]

Description of Additional Supplementary Files

File Name: Supplementary Data 1
Description: Significant differentially expressed genes in D24 or D17 versus D10.

File Name: Supplementary Data 2
Description: significant differentially expressed genes in actinonin treated hANOs versus untreated organoids on D24 and D17, respectively.

File Name: Supplementary Movie 1
Description: Time-lapse microscopy of hANOs during proliferation stage (D0 to D7).

File Name: Supplementary Movie 2
Description: Time-lapse microscopy of hANOs during differentiating stage (D11 to D17).

File Name: Supplementary Movie 3
Description: Time-lapse microscopy of hANOs during differentiated stage (D18 to D24).

File Name: Supplementary Movie 4
Description: Cilia beating patterns of hANOs during differentiated stage (D24).

File Name: Supplementary Movie 5
Description: No cilia beating evidence in hANOs treated with actinonin.
