# Supplementary material for: Human apical-out nasal organoids reveal an essential role of matrix metalloproteinases in airway epithelial differentiation
Source: Nat Commun. 2024 Jan 2;15:143. doi: 10.1038/s41467-023-44488-1 (PMC10762242; doi:10.1038/s41467-023-44488-1)
Supplement: Supplementary file 11 — Reporting Summary [file 41467_2023_44488_MOESM11_ESM.pdf]

nature portfolio

Corresponding author(s): Chun Liu, Chunwei Li

Last updated by author(s): Nov 25, 2023

Reporting Summary

Nature Portfolio wishes to improve the reproducibility of the work that we publish. This form provides structure for consistency and transparency in reporting. For further information on Nature Portfolio policies, see our Editorial Policies and the Editorial Policy Checklist.

Statistics

For all statistical analyses, confirm that the following items are present in the figure legend, table legend, main text, or Methods section.

n/a	Confirmed	
☐	☒	The exact sample size (*n*) for each experimental group/condition, given as a discrete number and unit of measurement
☐	☒	A statement on whether measurements were taken from distinct samples or whether the same sample was measured repeatedly
☐	☒	The statistical test(s) used AND whether they are one- or two-sided<br>*Only common tests should be described solely by name; describe more complex techniques in the Methods section.*
☒	☐	A description of all covariates tested
☐	☒	A description of any assumptions or corrections, such as tests of normality and adjustment for multiple comparisons
☐	☒	A full description of the statistical parameters including central tendency (e.g. means) or other basic estimates (e.g. regression coefficient) AND variation (e.g. standard deviation) or associated estimates of uncertainty (e.g. confidence intervals)
☐	☒	For null hypothesis testing, the test statistic (e.g. *F*, *t*, *r*) with confidence intervals, effect sizes, degrees of freedom and *P* value noted<br>*Give P values as exact values whenever suitable.*
☒	☐	For Bayesian analysis, information on the choice of priors and Markov chain Monte Carlo settings
☒	☐	For hierarchical and complex designs, identification of the appropriate level for tests and full reporting of outcomes
☒	☐	Estimates of effect sizes (e.g. Cohen's *d*, Pearson's *r*), indicating how they were calculated

Our web collection on statistics for biologists contains articles on many of the points above.

Software and code

Policy information about availability of computer code

Data collection	Gel pictures were obtained by using iPhone 8 plus (Apple, CA, USA). Bright field images were captured by IX51 inverted microscope (Olympus, Tokyo, Japan).Time-lapse videos were obtained by Lux3 FL live-cell imaging microscope (CytoSMART Technologies B.V., Eindhoven, The Netherlands). Cilia beat videos were obtained by by IX51 inverted microscope (Olympus, Tokyo, Japan) with the the Sisson-Ammons Video Analysis system(SAVA, Omaha, NE). 3D images were captured by using TCS SP8 DIVE multiphoton microscope (Leica, Mannheim, Germany) and reconstructed using a computer with LAS X software (Leica, Mannheim, Germany). Cytospin images ,Masson staining and IHC staining images were captured under DM4B upright microscope (Leica,Mannheim, Germany). RT-qPCR data were obtained by using Real-Time PCR System (Applied Biosystems, MA, USA). Luminex assay were acquired by using a Luminex MAGPIX system (Luminex, Austin, TX).MMP activity data were acquired by using VICTOR Nivo Multimode Plate Reader(Perkinelmer, MA, USA). For RNA-seq data, the library preparation was carried out by using NEBNext® UltraTM RNA Library Prep Kit for Illumina® (NEW ENGLAND BioLabs,MA, USA).cDNA libraries were sequenced on an Illumina Novaseq platform (Illumina,CA, USA).
Data analysis	ImageJ software version 1.52 (National Institutes of Health, Maryland,USA); Imaris 8.4 software version 8.4(Bitplane, Zurich, Switzerland); GraphPad Prism software version 8.0 (GraphPad Software, CA,USA); R version 4.0.3 was used to analysis RNA-seq data. RPKM counts were estimated using DEseq2 program (Bioconductor, https://www.bioconductor.org).).ClusterProfiler program was used to perform enrichment analysis.

For manuscripts utilizing custom algorithms or software that are central to the research but not yet described in published literature, software must be made available to editors and reviewers. We strongly encourage code deposition in a community repository (e.g. GitHub). See the Nature Portfolio guidelines for submitting code & software for further information.

Data

Policy information about availability of data

All manuscripts must include a data availability statement. This statement should provide the following information, where applicable:

- Accession codes, unique identifiers, or web links for publicly available datasets
- A description of any restrictions on data availability
- For clinical datasets or third party data, please ensure that the statement adheres to our policy

All RNA-seq data generated in this study are available on the GEO database (GSE编号+链接）.The data generated in this study are provided in the main article and its Supplementary Information file or available from the corresponding authors upon reasonable request. Source data are provided with this paper.

Research involving human participants, their data, or biological material

Policy information about studies with human participants or human data. See also policy information about sex, gender (identity/presentation), and sexual orientation and race, ethnicity and racism.

Reporting on sex and gender	Nasal mucosa from 23 male donors and 10 female donors was collected for cell culture or tissue staining experiments. No sex- or gender-based analysis was performed in this study.
Reporting on race, ethnicity, or other socially relevant groupings	Race, ethnicity and any other socially relevant grouping were not considered in this study.
Population characteristics	Nasal tissue was obtained from patients (aged between 18 and 65 years) with functional endoscopic sinus surgery or septoplasty surgery in the department of otolaryngology, The First Affiliated Hospital, Sun Yat-sen University (Guangzhou, China).
Recruitment	Biopsy specimens of nasal mucosa were obtained from patients with chronic rhinosinusitis,nasal septal deviation or nasal fracture who underwent functional endoscopic surgery at the First Affiliated Hospital, Sun Yat-sen University, Guangzhou, China. None of the patients had other systemic diseases and did not receive glucocorticoids, antibiotics, or both within 3 months before the study. None of the CRS patients and controls had immunodeficiencies, autoimmune diseases, tumor, or acute respiratory tract infection 1 month before inclusion. All subjects were carefully selected and only individuals who did not receive systemic glucocorticoid steroids, antibiotics for 3 months and/or nasal corticosteroids for 1 month before the surgery. There is no potential bias in this study as there is no different treatments or groups, samples are used for human nasal epithelial cell and organoid culture, or tissue staining.
Ethics oversight	Approval for this study was obtained from the Institutional Review Board of The First Affiliated Hospital, Sun Yat-sen University (Project number [2017] 256). All participants were given written informed consent.

Note that full information on the approval of the study protocol must also be provided in the manuscript.

Field-specific reporting

Please select the one below that is the best fit for your research. If you are not sure, read the appropriate sections before making your selection.

☒ Life sciences ☐ Behavioural & social sciences ☐ Ecological, evolutionary & environmental sciences

For a reference copy of the document with all sections, see nature.com/documents/nr-reporting-summary-flat.pdf

Life sciences study design

All studies must disclose on these points even when the disclosure is negative.

Sample size	Sample size for each experiment is indicated in the legend. No statistical methods were used to predetermine sample sizes. Sample size was chosen based on previous experiments and comparable. The reference of cellular experiments and tissue staining experiments sample size is: Tao Liu, et al. NOD-like receptor family, pyrin domain containing 3 (NLRP3) contributes to inflammation, pyroptosis, and mucin production in human airway epithelium on rhinovirus infection. J Allergy Clin Immunol.2019.144(3): 777-87 e9
Data exclusions	No data were exclued from the analysis.
Replication	All experimental findings were reproduced in multiple independent experiments. For each figure, the number of independent experiments or biological replicates is indicated in the corresponding legends. Gel dedagration picture and microscopy images were selected from a representative experiment and the number of independent repeats is clearly indicated in the figure legends.
Randomization	For organoid culture experiments, human nasal epithelial progenitor cells were split, plated in culture vessels, and then treated with normal medium or MMP inhibitor. Because control and treatment groups were derived from the same sample in each biological replication, no randomization could be performed. In tissue staining study, tissue were allocated for each group according to the diagnoses of the patients. Hence, no randomization could be performed, either.

Blinding	Investigators were blinded for analysis of histological specimens and immunofluorescent staining. In experiments without subjective estimation like luminex assays, qPCR, MMP activity and RNA-seq, investigators were unblinded since no bias would be introduced by the investigators. For other experiments, investigators were unblinded since all treatments performed in a parallel manner without the risk of bias in interpretation.

Reporting for specific materials, systems and methods

We require information from authors about some types of materials, experimental systems and methods used in many studies. Here, indicate whether each material, system or method listed is relevant to your study. If you are not sure if a list item applies to your research, read the appropriate section before selecting a response.

Materials & experimental systems

n/a	Involved in the study
☐	☒ Antibodies
☒	☐ Eukaryotic cell lines
☒	☐ Palaeontology and archaeology
☒	☐ Animals and other organisms
☐	☒ Clinical data
☒	☐ Dual use research of concern
☒	☐ Plants

Methods

n/a	Involved in the study
☒	☐ ChIP-seq
☒	☐ Flow cytometry
☒	☐ MRI-based neuroimaging

Antibodies

Antibodies used

Immunofluorescent (IF) staining:
anti- beta-Tubulin IV : Sigma-Aldrich, #T7941, clone ONS.1A6, 1:800
anti-ZO-1 : Sigma-Aldrich, #AB2272, polyclone, 1:500
anti-Mucin 5AC :Abcam, #ab198294, clone EPR16904, 1:600
anti-p63: Abcam, #ab735, clone 4A4, 1:100
anti-foxj1: Abcam, #ab235445, clone EPR21874, 1:1000
anti-Uteroglobin(SCGB1A1): Abcam, #ab307666, clone EPR27144-86, 1:1000
anti-DAPI: Roche, #10236276001, 1ug/ml
goat anti-mouse Alexa Fluor 488: Thermo Fisher Scientific, #A11029, 1:500
goat anti-rabbit Alexa Fluor 594: Thermo Fisher Scientific, #A11032, 1:500
The Collagen Hybridizing Peptide, 5-FAM Conjugate (F-CHP) was synthesized and provided by Prof. Yang Li from the fifth affiliated hospital of Sun Yat-Sen University (Zhuhai, China), and the work concentration of F-CHP is 5μM.

Immunohistochemistry(IHC) experiment:
anti-MMP7 : Abcam, #ab207299, clone EPR17888-71, 1:50
anti-MMP9: Abcam, # ab76003, , clone EP1254), 1:400
anti-MMP13: Abcam, #ab219620, rclone EPR21778, 1:20
anti-MMP10 : RD, #MAB910-SP, clone 110304, 1:100

Validation

For the application of F-CHP,please refer to Jeongmin Hwang, et al. In Situ Imaging of Tissue Remodeling with Collagen Hybridizing Peptides. ACS Nano.2017 Oct 24;11(10):9825-9835.The other antibodies in this study were commercially available and validated by manufacturers. Please refer to the data sheets from the vendor's website for technical info by searching the catalog number. provided.

Clinical data

Policy information about clinical studies
All manuscripts should comply with the ICMJEguidelines for publication of clinical research and a completedCONSORT checklist must be included with all submissions.

Clinical trial registration	No clinical trial was applied in the research.
Study protocol	The detail study protocol was indicated in the Experimental Section of the article.
Data collection	The clinical data were collected in the department of otolaryngology, The First Affiliated Hospital, Sun Yat-sen University (Guangzhou, China) from May 31, 2020 to June 30,2023.
Outcomes	The number of seeding cells and incubation time were pre-tested to develop the model of organoids.The concentration of the inhibitors were tested to predetermine the condition of stimulation.

Plants

Seed stocks	No seed stock was used in this study.
Novel plant genotypes	No palnt material was used in this study.
Authentication	No palnt material was used in this study.
